# Global Chemical Composition and Antioxidant and Anti-Tuberculosis Activities of Various Extracts of *Globularia alypum* L. (Globulariaceae) Leaves

**DOI:** 10.3390/molecules161210592

**Published:** 2011-12-19

**Authors:** Daycem Khlifi, Moktar Hamdi, Akrem El Hayouni, Sylvie Cazaux, Jean Pierre Souchard, François Couderc, Jalloul Bouajila

**Affiliations:** 1 Laboratoire des Interactions Moléculaires et Réactivité Chimique et Photochimique UMR CNRS 5623, University of Toulouse, University of Paul-Sabatier, 118 route de Narbonne, F-31062 Toulouse, France; 2 Laboratoire d’Ecologie et de Technologie Microbienne, Institut National des Sciences Appliquées et de la Technologie (INSAT), Université Carthage, B.P. 676, 1080 Tunis, Tunisia; 3 Laboratoire des Substances Biologiquement Actives, Centre de Biotechnologie à l’Ecopark de Borj-Cedria, BP-901, Hamman-Lif 2050, Tunisia

**Keywords:** *Globularia alypum* L., polyphenols, antioxidant activity, anti-tuberculosis activity

## Abstract

In this work, an evaluation of the biological activities of *Globularia alypum* L. extracts and their global chemical composition was realized. Extracts from *G. alypum* were obtained by two extraction methods. The composition of polyphenols (8.5–139.95 g gallic acid equivalent/Kg of dry mass), tannins (1.39–18.65 g catechin equivalent/Kg of dry mass), anthocyanins (8.17–70.69 mg cyanidin equivalent/Kg of dry mass) and flavonoids (0.31–19.28 g quercetin equivalent/Kg of dry mass) was evaluated. The samples were subjected to a screening for their antioxidant activities using the DPPH^●^ and ABTS^●^^+^ assays. For the first time, the anti-tuberculosis activity (H_37_Rv) for *G. alypum* was tested against *Mycobacterium* *tuberculosis*. The strongest antioxidant activity was obtained for the methanol extract (IC_50_ = 15.58 ± 0.168 mg/L) and the best anti-tuberculosis activity was obtained for the petroleum ether extract (IC_50_ = 77 mg/L). We have found a positive correlation between the total phenolics content and the antioxidant activity R^2^ = 0.88 (DPPH^●^) and R^2^ = 0.97 (ABTS^●^^+^). We have found also a positive correlation between the flavonoid content and the antioxidant activity R^2^ = 0.91 (DPPH^●^) and R^2^ = 0.91 (ABTS^●^^+^).

## 1. Introduction

Natural products have served as important sources of drugs since ancient times and a significant part of today’s drugs were somehow derived from natural sources. In recent years, a renewed interest in obtaining biologically active compounds from natural sources has been observed. There is an intense interest in plant polyphenols, as witnessed by numerous papers devoted to various aspects of these compounds [1]. Phenolics are antioxidants with redox properties, which allow them to act as reducing agents, hydrogen donors, and singlet oxygen quenchers [2]. They have also metal chelation properties [3]. Their significance for the human diet and antimicrobial activity has been recently established [4]. The antioxidant properties of these compounds are often claimed to be responsible for the protective effects of plant-based beverages against cardiovascular disease, certain forms of cancer and photosensitivity reactions [5]. Phenolics have also capacity to inhibit human immunodeficiency viral replication (HIV), human simplex virus (HSV), glucosyl transferases of *Streptococcus mutans* (dental carries), ascorbate auto-oxidation, cytotoxic effects, tumour promotion and xanthine, monoamine oxidases [6].

Antimycobacterial drugs cause unpleasant side effects and trigger changes in the antibiotic target, thereby reducing the efficacy of drug therapies. Mycobacteria have recently increased their virulence and tuberculosis (TB) is nowadays the most lethal infection in the World. From 1980 to 2005, 90 million cases of TB were reported worldwide to the World Health Organisation (WHO). The WHO stated "the global incidence of TB was estimated to be 136 cases per 100,000 population per year in 2005". In addition, the WHO region of the Americas and the WHO African region represent a total of 8.8 million new cases of TB and 1.6 million deaths from TB every year [7]. There were 9.5 million TB-related child deaths globally in 2006 [8]. Presently, one of the most important global health problems is the change of behaviour of TB such as resistance to anti-TB drugs and the influence of the HIV epidemic [7].

In order to search for new chemical compounds with potential antioxidant or anti-tuberculosis activities, we have investigated a medicinal plant, namely *Globularia alypum* L. The genus Globularia (Family: *Globulariaceae*) consists of plants which are herbs, chamaephytes or shrubs, common in the Mediterranean regions, Europe and North Africa (Tunisia, Morocco, Libya and Algeria). They are a rich source of phenolic compounds. *G. alypum* is commonly used in North African folk medicine. *G. alypum*, named locally as ‘zriga or Ain Larneb’ is a wild plant belonging to the Globulariaceae family. Skim *et al.* [9] confirmed the beneficial effects of *G. alypum* infusion against hypoglycemic agents. The hydromethanolic extract of *G. alypum* could thus be considered as a source of potential antioxidants and may promote the reasonable usage of this plant in food technology and processing as well as for medical use [10]. It was used as a hypoglycemic agent [11], for its fetotoxic potentials [12], immunosuppressive effects [13], laxative and purgative effects, stomachic role, and also in the treatment of cardiovascular and renal diseases [14].

The objectives of this study were: (i) to determine the chemical composition (polyphenols, tannins, flavonoids and anthocyannins) of various extracts from leaves of *G. alypum*; (ii) investigation of their antioxidant activity and, for the first time, anti-tuberculosis activity; and (iii) the study of possible correlations between chemical composition, anti-tuberculosis and antioxidant activities.

## 2. Results and Discussion

### 2.1. Chemical Composition

#### 2.1.1. Extraction Yields

Two methods were adopted to extract *G. alypum*. In the first extraction method (#1: one step extraction), 3:1 methanol/water was used and in the second extraction method (#2: sequential), different solvents of increasing polarity were employed: petroleum ether, dichloromethane, acetone, methanol/water (3:1) and water. Yields of different extracts obtained from leaves are presented in Table 1. The highest yield (42.4%) was recorded by the one-step method. For the second method the highest yields were achieved using the polar solvents: methanol (37%), followed by petroleum ether (15.6%), acetone (12.9%), dichloromethane (2.4%) and the lowest was with water (1.1%). Variation in the yields of various extracts can be attributed to the polarities of the different compounds in the leaves. Such differences have been reported in the literature [15]. There is no study in the literature which mentions the extraction yields using our methods or other methods.

**Table 1:** Extraction yields (%) and chemical composition of *Globularia alypum* L. extracts.

Samples		Yields (%)	Polyphenols (GAE) ^a^	Tannins (CE) ^a^	Flavonoids (QE) ^a^	Anthocyanins (C3GE) ^b^
Method (#1)	Methanol (75%)	42.4	116.89 ± 2.80	1.40 ± 0.06	18.20 ± 0.25	8.17 ± 0.70
Method (#2)	Petroleum ether	15.6	8.50 ± 0.10	5.73 ± 0.06	0.31 ± 0.02	nd
	Dichloromethane	2.4	41.19 ± 1.33	18.65 ± 0.11	0.34 ± 0.02	nd
	Acetone	12.9	109.46 ± 1.1	2.79 ± 0.07	17.08 ± 0.35	70.69 ± 1.53
	Methanol (75%)	37.0	139.95 ± 3.40	2.33 ± 0.06	19.29 ± 0.04	36.5 ± 2.01
	Water	1.1	55.10 ± 2.30	4.40 ± 0.06	1.33 ± 0.02	28.49 ± 3.55

^a^: g/Kg dry mass; ^b^: mg/Kg dry mass. Standard deviations (SD) did not exceed 5%. nd: not detected.

#### 2.1.2. Polyphenols Content

The amounts of the total polyphenols in the extracts fractions of *G. alypum* leaves are shown in Table 1. The amount of total phenolic contents in the different extracts ranged from 8.5 ± 0.1 to 139.95 ± 3.4 g GAE/Kg of dry mass. The highest amount of polyphenols was obtained by the methanolic extraction method (#2; 139.95 ± 3.4 g GAE/Kg of dry mass) and method #1 (116.89 ± 2.8 g GAE/Kg of dry mass), followed by acetone extract (109.46 ± 1.14 g GAE/Kg of dry mass). This latter result is due to the polarity of polyphenols and evidenced by the fact that in the petroleum ether, dichloromethane and water extracts there are fewer polyphenols.

These results showed that, for both methods, polyphenols content was strongly dependent on the solvent used. Polar fractions had more phenolics in them than had non-polar fractions. Hence, this could be used as an important descriptor to characterize the extracts of *G. alypum.* In another work, Djeridane *et al.* [16] obtained 21.54 g GAE/Kg of dry mass from a water-ethanol extract (30:70) of *G. alypum.* This extract was prepared according to the one-step extraction method. This result showed that the content of polyphenols was six times smaller than the one we found. There are no studies in the literature investigating the content of polyphenols in others members of the genus *Globularia.*

#### 2.1.3. Tannins Content

The amount of tannins in the dichloromethane leaves extracts were the highest (18.65 ± 0.11 g CE/Kg of dry mass) followed by petroleum ether extract (5.73 ± 0.06 g CE/Kg of dry mass). These results showed that tannins content was strongly dependent on the solvent used. Non-polar fractions had more tannins in them than polar fractions. This is the first study to record the tannins content of all extracts from *G. alypum.*

#### 2.1.4. Flavonoids Content

For the flavonoids, the highest quantity was found in the methanolic extract (method #2; 19.29 ± 0.04 g QE/Kg of dry mass) and with method #1 (18.20 ± 0.25 g QE/Kg of dry mass), followed by acetone extract (17.08 ± 0.35 g QE/Kg of dry mass). In another work, Djeridane *et al.* [16] obtained 4.54 g rutin/Kg of dry mass of aqueous/ethanol extract (30/70) from *G. alypum.* This result showed that the content of flavonoids was two times less important compared to what we found. There are no other studies in the literature that investigated the content of flavonoids in others members of the genus *Globularia.*

#### 2.1.5. Anthocyanins Content

For anthocyanins, the highest amount was obtained from the acetone extract (70.69 ± 1.53 mg C3GE/Kg of dry mass). Dichloromethane and petroleum ether contained no anthocyanins. These results showed that the anthocyanins content was strongly dependent on the solvents. Polar fractions had more anthocyanins in them than non-polar fractions. This the first study to record the anthocyanins content of different extracts from *G. alypum.*

### 2.2. Antioxidant Activity

**Figure 1:**
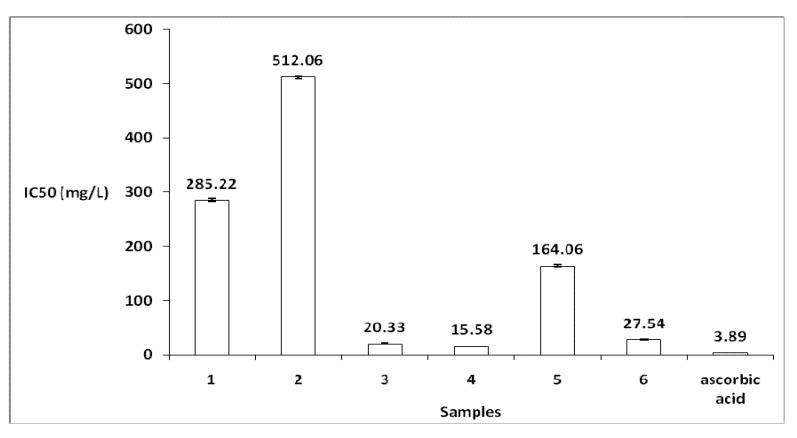
Antioxidant activity by DPPH^●^ assay. 1: petroleum ether extract method (#2); 2: dichloromethane extract method (#2); 3: acetone extract method (#2); 4: methanol/water (3/1) extract method (#2); 5: water extract method (#2) and 6: methanol/water (3/1) extract method (#1). Ascorbic acid was used as reference standard. Standard deviations (SD) did not exceed 5%.

**Figure 2:**
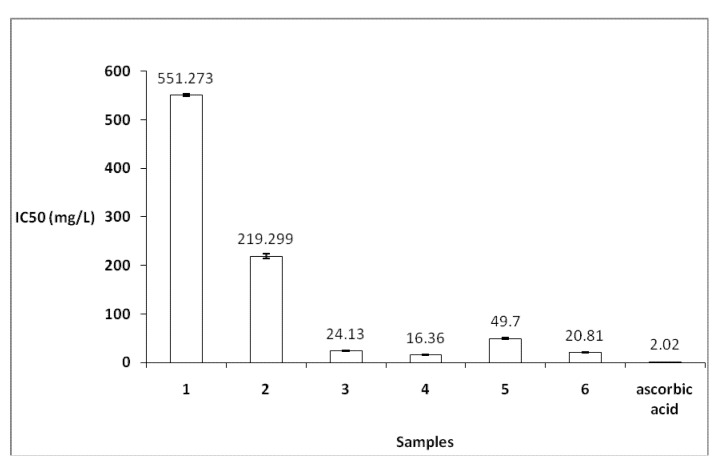
Antioxidant activity by ABTS^●^^+^ assay. 1: petroleum ether extract method (#2); 2: dichloromethane extract method (#2); 3: acetone extract method (#2); 4: methanol/water (3/1) extract method (#2); 5: water extract method (#2) and 6: methanol/water (3/1) extract method (#1). Ascorbic acid was used as reference standard. Standard deviations (SD) did not exceed 5%.

Antioxidant activity of the various *G. alypum* extracts have been determined by two different test systems, namely the DPPH^●^ and ABTS^●^^+^ assays. All data are presented in Figure 1 and Figure 2.

#### 2.2.1. DPPH^●^ Assay

The DPPH^●^ free radical method determines the antiradical power of antioxidants. The antioxidant activity of the methanol extract was superior to that of all other samples tested, with an IC_50_ value of 15.58 ± 0.17 mg/L, followed by the acetone extract (20.33 ± 0.61 mg/L). On the other hand, extracts prepared with petroleum ether or dichloromethane presented poor radical-scavenging activity (285.22 ± 2.77 mg/L and 512.05 ± 2.061 mg/L, respectively) compared to vitamin C. We can deduce, the methanol extract presented important activity comparable to vitamin C (3.89 ± 0.04 mg/L). As seen on Figure 1, it can be concluded that the extracts obtained using high polarity solvents were considerably more effective radical- scavengers than were those using low polarity solvents. Change in solvent polarity alters its ability to dissolve a selected group of antioxidant compounds and influences activity estimation.

In other works, Es-Safi *et al.* [10] have isolated a phenolic compound, 6-hydroxy-luteolin-7-*O*-laminaribioside, from the aerial parts of *G. alypum*,which displays an important antioxidant activity with IC_50_ = 1.76 mg/L; they used the BHT (butylated hydroxytoluene) as the positive control with IC_50_ = 8.81 mg/L.

Antioxidant activity has not been studied for *G. alypum* extract growing in Morocco. The difference between our results and Es-Safi *et al.* [10] results can be attributed to the different plant parts used we have used the leaves and they are used the aerial part and also to the different methods of extraction we have used the Soxhlet, while they were used a simple maceration and finally, the climate differences between Tunisia and Morocco, geographical origin, harvesting time and growing conditions.

#### 2.2.2. ABTS^●+^ Assay

A good antioxidant activity (Figure 2) value was presented by the methanol extracts [IC_50_ = 16.36 mg/L method (#1) and 20.81 mg/L method (#2)], followed by the acetone extract (IC_50_ = 24.13 mg/L). This result confirms the agreement between the solvent polarity and antioxidant activity where the methanol extract presented higher total phenolic quantity (139.95 ± 3.4 g GAE/Kg of dry material).

The antioxidant activity results showed some differences between the two tests. The ABTS^●^^+^ reactions involve electron transfer and take place at a much faster rate compared to DPPH^●^ [17] whose degree of discoloration is attributed to the hydrogen donating ability to tested compounds.

### 2.3. Anti-Tuberculosis Activity

In the present study, the anti-tuberculosis activity of the petroleum ether extract was superior to that of all samples tested, with IC_50_ = 77 mg/L (Table 2), followed by the dichloromethane extract (IC_50_ = 98 mg/L). This is an important activity since an IC_50_ were less than 100 mg/L [19]. The recent study of Kishore *et al.* [18] showed that alkaloids have anti-tubercular potential. In another work, Lall and Meyer [19] found that the, diospyrin (bisnaphthoquinone) isolated from *Euclea natalensis* has activity against *Mycobacterum tuberculosis* with MIC = 100 mg/L.

The petroleum ether extract had a low content of polyphenols (8.5 ± 0.10 g GAE/Kg of dry mass) followed by the dichlorometane extract (41.19 ± 1.33 g GAE/Kg of dry mass), indicating that the anti-tuberculosis activity was not due to phenolics, and other compounds must be responsible for this activity. This is the first study to investigate the anti-tuberculosis activity of a member of the genus *Globularia.*

**Table 2:** Anti-tuberculosis activity of *Globularia alypum* L.

		CMI mg/L				IC_50_ mg/L (PRISM)			
Test		1	2	3	Average	1	2	3	Average
Method (#1)	Methanol (75%)	>500	>500	>500	>500	nd	nd	nd	nd
Method (#2)	Petroleum ether	125	250	250	250 ± 32	41	132	88	77 ± 11
	Dichloromethane	250	500	250	250 ± 41	36	154	123	98 ± 13
	Acetone	250	>500	>500	>500	135	nd	nd	nd
	Methanol (75%)	>500	>500	>500	>500	nd	nd	nd	nd
	Water	>500	>500	>500	>500	nd	nd	nd	nd

nd: Not Detected.

### 2.4. Correlations

We observed that the content of phenolics in the extracts correlates with their antioxidant activity, the R^2^ correlation coefficients between the DPPH^●^ and ABTS^●^^+^ assay data and total phenolics were 0.87 and 0.96, respectively. This result suggests that between 87% and 96% of the antioxidant capacity (DPPH^●^ and ABTS^●^^+^, respectively) of the extracts is due to the contribution of phenolics. We also observed a good correlation between the DPPH^●^ and ABTS^●^^+^ assays and flavonoids contents (0.91 and 0.92 respectively).

This is the first correlation obtained for different extracts of this plant between polyphenols or flavonoids and antioxidant activity, so this correlation can guide the search for molecules responsible for the antioxidant activity. It confirms that phenolic compounds contribute to the radical scavenging activity of *G. alypum* extracts*.*

Several studies have focused on the relationship between the antioxidant activity of phenolic compounds as hydrogen donating free radical scavengers and their chemical structure. It has been shown that the presence of the –CH=CH-COOH group in the hydroxylated cinnamates ensures greater H-donating ability and subsequent radical stabilization than the carboxylate group in the hydroxy benzoates [20]. There was no correlation between anti-tuberculosis activity and the total phenolics.

## 3. Experimental

### 3.1. Collection of Plant Material

The fresh leaves of *G. alypum L* were collected in January 2009 from the central area of Tunisia, to be precise from the Sidi Bouzid region. Specimens were identified at the Department of Botany, National Institute of Applied Sciences and Technology (INSAT, Tunis) and voucher specimens (number: GA1) were deposited at the Herbarium of the Department of Botany in the cited institute.

### 3.2. Extraction

The fresh leaves were air dried in the shade at room temperature, and the dry leaves were then powdered. Two extraction methods were adopted for these powders. In the first extraction method (#1: one step extraction), powder (50 g) was extracted in a Soxhlet system with methanol-water (3:1, 500 mL) for 48 h at 65 °C. In the second extraction method (#2: sequential), powder (50 g) was also extracted in a Soxhlet system with 500 mL of different solvents of increasing polarity: petroleum ether (6 h at 40 °C), dichloromethane (6 h at 40 °C), acetone (6 h at 56 °C), methanol-water (3:1) (6 h at 65 °C) and water (6 h at 100 °C). All organic extracts were concentrated by rotary evaporation under vacuum at 35 °C. The water extract was freeze-dried.

### 3.3. Determination of Total Phenolic Compounds by the Folin-Ciocalteu Method

The polyphenols of each extract were determined by the Folin-Ciocalteu method [21]. A diluted solution of each extract (0.5 mL) was mixed with Folin Ciocalteu reagent (0.2 N, 2.5 mL). This mixture rest at room temperature for 5 min and then sodium carbonate solution (75 g/L in water, 2 mL) was added. After 1h of incubation, the absorbance was measured at 765 nm against water blank. A standard calibration curve was plotted using gallic acid (0–300 mg/L). The results were expressed as mg of gallic acid equivalent (GAE)/Kg of dry mass.

### 3.4. Condensed Tannin Content

Catechins and proanthocyanidins reactive to vanillin were analyzed by the vanillin method [22,23]. One milliliter of each extract solution was placed in a test tube and vanillin (1% in 7 M H_2_SO_4_, 2 mL) in an ice bath and then incubated at 25 °C. After 15 min, the absorbance of the solution was read at 500 nm. The concentrations were calculated as g catechin equivalent (CE)/Kg dry mass from a calibration curve.

### 3.5. Total Flavonoids Determination

The total flavonoids were estimated according to the Dowd method as adapted by Arvouret-Grand *et al.* [24]. A diluted solution (4 mL) of each extract was mixed with a 2% solution of aluminium trichloride (AlCl_3_) in methanol (4 mL). The absorbance was read at 415 nm after 15 min against a blank sample consisting of a methanol (4 mL) and extract (4 mL) without AlCl_3_. Quercetin was used as reference compound to produce the standard curve, and the results were expressed as g of quercetin equivalent (QE)/Kg of dry mass.

### 3.6. Determination of Total Anthocyanin Content

Total anthocyanin content was measured by the pH differential method, as described by Cheng and Breen [25]. Briefly, absorbance of the extract was measured at 510 and 700 nm in buffers at pH 1.0 (hydrochloric acid-potassium chloride, 0.2 M) and 4.5 (acetic acid-sodium acetate, 1 M). The wavelength reading was performed after 15 min of incubation. Anthocyanin content was calculated using a molar extinction coefficient (ε) of 29,600 (cyanidin-3-glucoside) and absorbance of A = [(A_510_ − A_700_) _pH 1.0_ − (A_510_ − A_700_) _pH 4.5_]. Results were expressed as mg cyanidin-3-glucoside equivalent (C3GE) /Kg of dry mass.

### 3.7. DPPH^●^ Free Radical Scavenging Activity

Antioxidant scavenging activity was studied using the 1,1-diphenyl-2-picrylhydrazyl free radical (DPPH^●^) as described by Blois [26] with some modifications; various dilutions of the test materials (ascorbic acid or plant extracts, 1.5 mL) were mixed with a 0.2 mM methanolic DPPH^●^ solution (1.5 mL). After an incubation period of 30 min at 25 °C, the absorbance at 520 nm were recorded as A_(sample)_. A_(blank)_ experiment was also carried out applying the same procedure to a solution without the test material and the absorbance was recorded. The free radical scavenging activity of each solution was then calculated as percent inhibition according to the following equation:

% inhibition = 100 × [(A_(blank)_ − A_(sample)_) / A_(blank)_]

Antioxidant activity extracts was expressed as IC_50_, defined as the concentration of the test material required to cause a 50% decrease in initial DPPH^●^ concentration. Values were estimated using linear regression. Ascorbic acid was used as a standard.

### 3.8. ABTS^●+^ Radical-Scavenging Test

The radical scavenging capacity of the samples for the ABTS^●^^+^ (2,2’-azinobis-3-ethylbenzo- thiazoline-6-sulphonate) was determined as described by Re *et al.* [27]. ABTS^●^^+^ was generated by mixing a 7 mM solution of ABTS^●^^+^ at pH 7.4 (5 mM NaH_2_PO_4_, 5 mM Na_2_HPO_4_ and 154 mM NaCl) with 2.5 mM potassium persulfate (final concentration) followed by storage in the dark at room temperature for 16 h before use. The mixture was diluted with ethanol to give an absorbance of 0.70 ± 0.02 units at 734 nm using spectrophotometer. For each sample, diluted methanol solution of the sample (100 μL) was allowed to react with fresh ABTS^●^^+^ solution (900 μL), and then the absorbance was measured 6 min after initial mixing. Ascorbic acid was used as a standard and the capacity of free radical scavenging was expressed by IC_50_ (mg/L) values calculated denote the concentration required to scavenge 50% of ABTS^●^^+^. The capacity of free radical scavenging IC_50_ was determined using the same previously used equation for the DPPH^●^ method.

### 3.9. Anti-Tuberculosis Activity

The susceptibility of *M. tuberculosis* strain H_37_Rv to all extracts was evaluated using a colorimetric micro assay based on the reduction of MTT (3-(4,5-dimethylthiazol-2-yl)-2,5-diphenyltetrazolium bromide) to formazan by metabolically active cells. Briefly, serial two fold dilutions of each extract solubilised in DMSO were prepared in 7H9 broth (Middlebrook 7H9 broth base (Difco)) using 96-well microtiter plates and 100 µL of *M. tuberculosis* H37Rv suspension in 7H9 broth were added to each well. After 6 days incubation, MTT was added (50 µL, 1 mg/mL). After one day incubation, solubilisation buffer was added to each well. The optical densities were measured at 570 nm. The MIC was determined as the lowest concentration of drug that inhibited bacterial growth (absorbance from untreated bacilli was taken as a control for growth). When possible, IC_50_ was determined by using the GraphPad Prism 5.0 Software [28].

### 3.10. Statistical Analysis

All data were expressed as means ± standard deviations of triplicate measurements. The confidence limits were set at P < 0.05. Correlations were carried out using the correlation and regression in the EXEL program.

## 4. Conclusions

*G. alypum* extracts were investigated for their chemical composition, antioxidant and anti-tuberculosis activities. Our results clearly showed that the extracts were rich in polyphenols, with levels up to 6 times higher than previously reported in the literature. As observed, we can conclude that methanolic extract with higher antioxidant capacity (IC_50_ = 15.58 mg/L by DPPH^●^ assay and IC_50_ = 16.36 mg/L by ABTS^●^^+^ assay) could be considered a potential natural antioxidant alternative for use in the food industry along with their possible applications in pharmaceutical industry for the prevention or treatment caused by microorganisms and free radicals. Further studies are necessary to assess the methanolic extraction of the compounds responsible for the antioxidant activity which could have far more effective capacity than vitamin C and might be used as new antioxidants and alternatives to synthetic antioxidants. This is the first study to investigate the anti-tuberculosis activity of *G. alypum* and the members of the genus *Globularia.* To confirm the anti-tuberculosis activity of this plant, we began purification of petroleum ether extract (IC_50_anti-tuberculosis = 77 mg/L) to identify the compounds other than polyphenols in the extract responsible for the anti-tuberculosis activity and to purify them to determine the target molecule.
